# New Evidence of Model Crop *Brassica napus* L. in Soil Clean-Up: Comparison of Tolerance and Accumulation of Lead and Cadmium

**DOI:** 10.3390/plants10102051

**Published:** 2021-09-29

**Authors:** Mihaela Rosca, Petronela Cozma, Mariana Minut, Raluca-Maria Hlihor, Camelia Bețianu, Mariana Diaconu, Maria Gavrilescu

**Affiliations:** 1 Department of Environmental Engineering and Management, “Cristofor Simionescu” Faculty of Chemical Engineering and Environmental Protection, “Gheorghe Asachi” Technical University of Iasi, 73 Prof. D. Mangeron Blvd., 700050 Iasi, Romania; mihaelarosca@uaiasi.ro (M.R.); minut.mariana@yahoo.com (M.M.); raluca.hlihor@uaiasi.ro (R.-M.H.); betianuc@tuiasi.ro (C.B.); mdiaconu@tuiasi.ro (M.D.); 2 Department of Horticultural Technologies, Faculty of Horticulture, “Ion Ionescu de la Brad” Iasi University of Life Sciences, 3 Mihail Sadoveanu Alley, 700490 Iasi, Romania; 3 Academy of Romanian Scientists, 3 Ilfov Street, 050094 Bucharest, Romania

**Keywords:** germination, phytoremediation, photosynthetic pigments, rapeseed, translocation factor

## Abstract

The potential of the model crop *Brassica napus* L. (rapeseed) for the phytoremediation of soils polluted with metals was investigated at laboratory scale. The first step consists in the evaluation of the seed germination and growth of the *Brassica napus* L. plant in a controlled environment, followed by the determination of the photosynthetic pigments content represented by chlorophyll a and b and carotenoids. The degree of metal accumulation in rapeseed has been evaluated by the bioaccumulation factor (BAC), the bioconcentration factor (BCF) and the translocation factor (TF). Phytotoxicity tests were performed in Petri dishes with filter papers moistened with metal solutions in the range of 0 to 300 mg/L Pb(II) or Cd(II). At the highest concentration of the lead and cadmium treatments (300 mg/L), *B. napus* L. showed the lowest germination degree (56.67% and 43.33%, respectively). According to Tukey test results, Pb(II) concentrations of up to 300 mg/L do not significantly affect the length of the hypocotyls, whereas, in the case of Cd(II), the mean of the radicle and hypocotyl lengths of the seedlings are significantly affected compared to the mean of the control. In soil pot experiments, important changes have been obtained in the pigment content, especially in the case of cadmium. For both metals and for each treatment (100 to 1500 mg/kg Pb(II) and 1 to 30 mg/kg Cd(II)), a TF < 1 indicates an ineffective metal transfer from root to shoot. Finally, rapeseed can be considered a tolerant plant and a suitable candidate for Pb(II) and Cd(II) accumulation and for the phytostabilization of contaminated soil under the experimental conditions adopted in the present study.

## 1. Introduction

**Figure 1:**
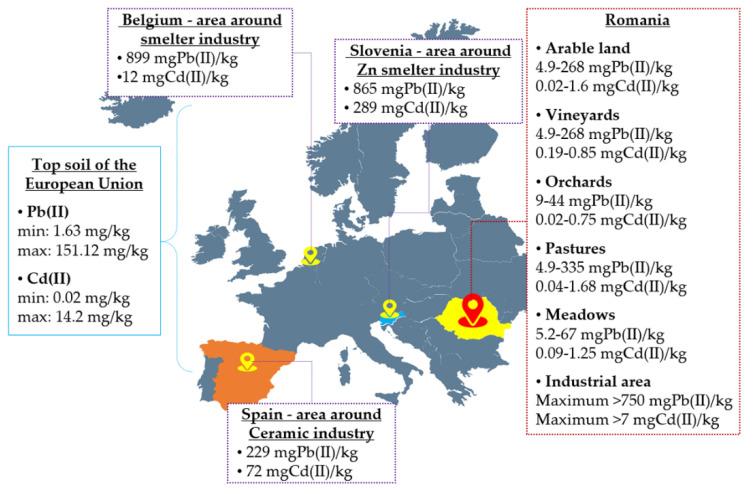
Cadmium and lead levels in soil in different areas of Europe and Romania (data in the Figure are selected from the sources [4,5,6]).

The contamination of different environmental compartments by potentially toxic elements (PTEs) (metals or non-metals), especially soil, increased starting with the 20th century, along with the industrial revolution, technological developments, population growth and the consumer use of several materials containing metals (e.g., agricultural amendments, mining and military activities, smelting industry, plastic manufacturing, etc.) [1,2]. Currently, issues are being raised on urban soil pollution with metals that typically contain higher amounts of contaminants compared to rural soils, taking into account their concentrations, distribution and impact at a global, local and regional level [3]. The LUCAS Topsoil Survey of the European Union represents the basic data regarding the distribution of metals, especially in the topsoil of Europe [4] (Figure 1). Based on the available maps of the metal concentration in topsoil and different industrial areas, institutions responsible for soil quality may control the potential threat from metals by their continuous monitoring [4].

Among different PTEs, lead and cadmium are seen as serious environmental pollutants due to their toxic effect on human health, plants, animals and microorganisms [7], and their persistence and bioaccumulative and non-biodegradable properties [8]. Lead is generally ranked as the main pollutant in the environment, among other metals [9]. The International Agency for Research on Cancer (IARC) classified cadmium in the first category as a carcinogen (Group 1—which means that there is sufficient evidence for its carcinogenicity in humans) [10,11,12] and lead in the Group 2B as a carcinogen (which means that it is probably carcinogenic to humans, since there is some evidence for an increased risk of lung and stomach cancer to human populations) [13]. Further, lead and cadmium are on the first and seventh place, respectively, in the substance priority list reported by the United States Agency for Toxic Substances and Disease Registry (ATSDR) [14,15].

Cadmium, as a carcinogen, once absorbed in the human body, accumulates in the liver, kidney, bones and muscle tissues, posing a serious risk to human health [16,17,18]. Cd, which is a transition metal, is a non-essential element for plants. Once it reaches the soil, Cd can be easily taken up by plants and accumulated in different parts of them [7,19,20]. Further, along with their accumulation in plants, the toxic elements enter the food chains [18,21]. In the presence of Cd stress, plants growth is often inhibited. Other known effects are: leaf chlorosis, disorder of nutrient uptake and mineral absorption, reduction of biomass production, changes in the chlorophyll content, photosynthesis inhibition and promoting the production of reactive oxygen species (ROS), including hydrogen peroxide (H_2_O_2_) [16,22,23,24,25,26,27]. 

Lead is a non-essential element in the metabolic process, compromising soil productivity and being toxic for plants, microorganisms and animals, even at low concentrations. Brain damage and retardation are the main harmful effects of lead to human health [28,29]. In the presence of Pb stress, plants growth and seeds germination are also affected. Pb has been reported to induce oxidative stress, cause a reduction in the chlorophyll content and leaf chlorosis, inhibit root and shoot growth as well as enzymatic activities and photosynthesis [9,30].

Consequently, it is clear that the accumulation of metals in agricultural soils is a serious problem for human health and safe food production. Thus, the treatment of polluted soils is absolutely necessary to solve current problems in agricultural production, at least. Among different methods developed to remove PTEs from contaminated soils, phytoremediation has gained extensive attention from specialists due to some important advantages it possesses, such as: low costs, effectiveness, simplicity and ease of implementation as an environmentally friendly technique [1,2,7,22,31]. Phytoremediation involves different mechanisms, such as phytoextraction, phytovolatilization, phytostabilization, phytofiltration, phytodegradation and/or rhizodegradation [1]. Phytoextraction (which is the main mechanism in metals removal by plants, followed by phytostabilization) involves the withdrawal of pollutants from soil by the roots followed by their uptake into the aerial plant tissues (leaves and shoots) until the metal concentration is reduced to an acceptable limit [32]. The resulting biomass can be further processed through incineration or drying, followed by metal recovery by bioleaching [1].

The successful application of phytoremediation technology depends on several factors [31]. First of all, plants are known to be sensitive to contaminants [33]. Secondly, not all of the plants have (hyper)accumulation properties so as to uptake and sequester the toxic metals, such as Cd and Pb, in their shoots [31]. However, limited hyperaccumulators have been reported (around 500 species), from which, more than 75% are Ni hyperaccumulators [23], and a relatively low number of plants were reported as Cd and Pb hyperaccumulators [31,34]. This is because hyperaccumulators are metal-selective plants, being difficult to grow and harvest and only being able to be used in their natural habitats, while their slow growth and low resulting biomass may limit the rate of metal removal [22,23]. For example, lead can be tolerated only by plants with a high biomass, which are able to accumulate more than 1% lead in their shoot [35].

Different plant species of cereal crops (corn, wheat, rye, bean)—*Zea mays* L. [24], *Triticum aestivum* L. [36,37], *Secale cereale* L. [36], *Phaseolus vulgaris* L. [37]; energy crops or herbaceous plants (soybean, sunflower, rapeseed, white mustard, Indian mustard, turnip)—*Glycine max* L. [2], *Helianthus annus* L. [1,38], *Brassica napus oleifera* L. [16,38], *Sinapis alba* L. or *Brassica alba* [21,25,36], *Brassica napus* L. [39,40,41,42] *Brassica juncea* L. [43,44,45], *Brassica campestris* [45]; ornamental plants—*Calendula officinalis*, *Althaea rosea*, *Impatiens balsamina* [34] have been reported as suitable for the remediation of Cd or Pb-contaminated soils.

We can observe that several studies explored *Brassica* species (*Brassica napus* L.—rapeseed; *Brassica juncea* L.—Indian mustard, brown mustard; *Brassica alba* L.—white mustard; *Brassica campestris* or *Brassica rapa*—Chinese cabbage, turnip) as potential phytoextractor plants that are able to accumulate high concentrations of toxic metals, such as Cd and Pb (and other metals, such as Cr, Cu, Ni, Zn) [41,46]. However, in this regard, some contradictory opinions exist in literature, since different soil experiments can support the phytoextraction potential of rapeseed [41,47] whereas others cannot [16,43]. Even so, the experiments in soil are very complex. First of all, the efficiency of metal extraction and accumulation by plants depend on the soil type, soil matrix, physical–chemical properties of the soil, mobility and concentration of metals in soils, climatic conditions that can be very different depending on the studied area [16,43]. Secondly, some researchers used in their experiments soil from different regions contaminated over years due to anthropogenic activity, whereas others used artificially contaminated soils and developed the experiments after a few days or weeks, which were required in order to attain an equilibrium state. Finally, other parameters, such as the plant phenology at contamination or harvesting and soil fertilization or lack of fertilization will all pose a huge impact on the experimental results [46].

Cadmium and lead were selected for the present study due to their high concentrations in EU soils related to industrial development (Figure 1). As mentioned above, Cd and Pb are considered carcinogenic and probably carcinogenic, respectively, to humans. The concentration ranges selected for this study (Cd: 1–30 mg/kg soil and Pb: 100–1500 mg/kg soil) were established, taking into account cadmium and lead levels in different areas of Romanian and EU soils (Figure 1) and also considering the alert and intervention thresholds established by the Order no. 756 from 3 November 1997 for the approval of the regulation on the assessment of environmental pollution in Romania [48] (Table 1). According to the Ministry of the Environment of Finland [49], the threshold value for Cd and Pb in soil is 1 mg/kg and respectively 60 mg/kg, whereas permissible limits for Cd and Pb are 10 mg/kg (on the basis of ecological risk) and 200 mg/kg (on the basis of health risk), respectively. WHO standard permissible limits for metals in soil are 0.8 mg/kg for Cd and 85 mg/kg for Pb, whereas intervention values of soil for Cd and Pb are 12 mg/kg and 530 mg/kg, respectively [50]. Finally, it can be concluded that metal regulatory limits in agricultural soils may differ worldwide, which is due to different standards of human health and ecological risks [51].

For this study, we selected the specie of *Brassica napus* L. ssp. *oleifera* Metzg, cultivated in Romania especially for its seeds that contain 44.5–45.8% oil used for various purposes, such as vegetable oil for human nutrition and for the production of margarine, which is a processed food. After the extraction of oils, the resulting by-products are used in animal feed [52]. For example, in 2021, there was the highest average production of rapeseed per hectare (3022 kg/ha equivalent to a quantity of 1.33 million tons) since 2007 until now. Rapeseed (*Brassica napus* L.) was taken for this work as a model crop for the phytoremediation of pollutes soils, since it is a well-known metal-tolerant plant. Even if they are considered as a non-hyperaccumulating species, these plants are able to grow quickly and produce a high amount of biomass, which are important elements in phytoremediation [53,54]. It can be said that, due to the production of a large amount of biomass, the overall accumulation of phytoextractor plants is close to that of hyperaccumulators [55]. Moreover, the plants of the *Brasicaceae* family are able to produce high levels of glutathione and phytochelatins, which are significant metabolites, assuring plant growth under metal stress conditions [53].

In this regard, the objective of our paper is to study the effect of cadmium and lead on: (i) seed germination and the growth of the *Brassica napus* L. plant in a controlled environment; (ii) the photosynthetic pigment content represented by chlorophyll a and b and carotenoids; and (iii) the phytoremediation potential of rapeseed by the determination of the accumulation and distribution of Cd and Pb in various plant parts. The phytoremediation potential of the rapeseed plantby phytoextraction or phytostabilization was evaluated by three factors: bioaccumulation factor (BAC), bioconcentration factor (BCF) and translocation factor (TF).

**Figure 2:**
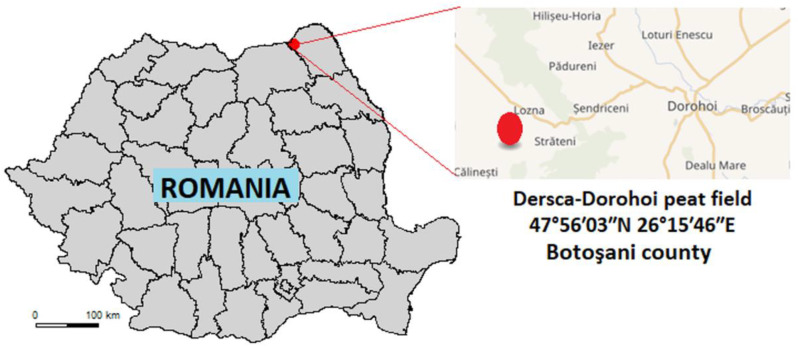
Dersca–Dorohoi peat field location map.

Finally, this study aims to provide new evidence for plant tolerance used in phytoremediation by adapting plant-soil-contaminant interactions to environmental conditions in Romania. Due to its wide spread in Romania and its ability to retain metals, the potential of rapeseed to decontaminate soils can be largely exploited. In this regard, we selected a type of Romanian soil taken from the Dersca–Dorohoi peat field in the N-E part of Romania, which was artificially contaminated with the level of metals appropriated to Romanian agricultural and industrial soils (Figure 2). To the best of our knowledge, there is little research developed in Romania in this field and no research has considered the rapeseed plant. For example, Damian et al. [56] used the *Sinapis alba* L. (white mustard) plant as a bioindicator to revegetate the metal-polluted soil from the metallurgical plant Romplumb Baia Mare, Romania. Stancu [57] tested different plants (*Rumex acetosella*, *Rubus caesius*, *Crataegus monogyna*, *Betula pendula* and *Agrostis capillaris*) in her PhD thesis to remediate a contaminated area from the Metaliferi Mountains, which are known as the main gold production center in Romania, with great significance in mining. Recently, Diaconu et al. [58] used the *Lepidium sativum* plant and two microorganisms (*Azotobacter chroococcum* and *Pichia spas*) as models for studying the toxicity of metals (Cd(II) and Cr(VI)) on their development as a preliminary approach for soil phytoremediation. They used three types of soils: two soils from the Iasi area and one soil type from the Gurghiu Mountains, Romania. Tenea et al. [59] developed a laboratory study to evaluate the potential of *Sinapis alba* L. to tolerate cadmium in the presence of essential metals. They used a peat soil and humus for garden plants as a soil substrate.

Our final results showed that rapeseed had the ability to retain Cd and Pb in their roots (under the conditions tested), which may contribute to a reduced accumulation of Cd and Pb in the crop plants, and, as a consequence, can be of great interest to human health, especially when it is used for the production of vegetable oil for human nutrition.

## 2. Materials and Methods

### 2.1. Phytotoxicity Assay

Phytotoxicity tests were performed on filter paper in Petri dishes with 9 cm diameter, in sterile conditions. The test plates together with Whatman filter paper were previously sterilized at 121 °C in autoclave for 20 min and further dried in oven at 35 °C. Seeds of *Brassica napus* L. ssp. *oleifera* Metzg (rapeseed) were sterilized with 96% alcohol for 20 s, followed by 20% NaClO solution for 20 min and washed seven times with sterile deionized water (adapted upon Lee et al. [31]). In each Petri dish, 10 seeds of *Brassica napus* L. and 3 mL of sterile distillated water (as a control probe) or synthetic solutions of 25, 50, 100, 150, 200, 250 and 300 mg/L Pb(II) or Cd(II) were placed. The experiments were performed in triplicate. Seed germination was carried out at 23 ± 2 °C under laboratory conditions and the filter paper was moistened daily with sterile deionized water to maintain the optimum humidity for germination and growth. The phytotoxicity tests lasted 5 days. Other studies consider that 4 days of germination are enough to obtain an 80 to 100% germination of *B. napus* [60].

### 2.2. Statistical Analysis

At the end of germination stage, the number of germinated seeds was counted and lengths of the radicle and hypocotyls were measured using a slide rule. The germination rate (GD) for each replicate and metal concentration was calculated with the Equation (1):

GD (%) = (Number of germinated seeds/Number of total seeds) × 100(1)

The variation in length of the radicle/hypocotyls and germination rate were evaluated by one-way analysis of variance (ANOVA) and the significant differences among lead and cadmium treatments were analyzed using HSD test. Data were tested at significant levels of *p* < 0.05 using Minitab 17 Statistical Software, a statistics package developed at the Pennsylvania State University, USA.

### 2.3. Plant Growth in Contaminated Soil

*Brassica napus* L. plants were grown during 25 days in 50 mL Falcon tubes, filled with 35 g sterile contaminated peat soil. The soil used in the experiments was a universal substrate composed of peat soil and humus for garden plants (S.C. FLORISOL PRODUCT S.R.L), which, according to the supplier, was taken from the Dersca–Dorohoi peat field and contains 192 ppm P, 1350 ppm K, 410 ppm N and pH 6.5–7. The soil was previously dried at 105 °C for 12 h and artificially contaminated with lead and cadmium ions using PbCl_2_ and CdCl_2_ solutions of known concentration. The soil from each tube was moistened with 25 mL metal solution, which contained the appropriate amount of metal for ensuring 100, 200, 500, 750, 1000 and 1500 mg/kg of Pb(II) per kg of soil and 1, 5, 10, 15, 20 and 30 mg/kg of Cd(II) per kg of soil, respectively. The contaminated soil was kept moist for a week and then three seeds were sown in each tube. After seedling emergence, when plants reached a maximum of 2 cm in length, only one plant was kept in each tube. All of the experiments were carried out under laboratory conditions, between 1st and 25th of June 2019. 

### 2.4. Determination of Photosynthetic Pigments in Plants 

The chlorophyll a and b and carotenoid pigments in plant leaves were determined by spectrophotometric method, using 96% ethanol as solvent. An amount of 300 mg fresh leaf tissue was crushed, mixed with 15 mL ethanol and kept in the dark at 4 °C for 48 h. The absorbance of extracts, after filtration with 0.5 µm PTFE filters, was measured at 664 nm, 649 nm and 470 nm wavelengths, using 3.5 mL quartz cuvettes and a UV-VIS spectrophotometer (T60, PG Instruments, Leicestershire, UK). Based on absorbance values, the content of chlorophyll a and b and carotenoids in plant leaves were calculated using the Equations proposed by Lichtenthaler [61]:

*Chlorophyll a* (mg/mL) = 13.36 × *A*_664_ − 5.19 × *A*_649_ (2)

*Chlorophyll b* (mg/mL) = 27.43 × *A*_649_ − 8.12 × *A*_664_ (3)

*Carotenoids* (mg/mL) = (1000 × *A*_470_ − 2.13 × *Chlorophyll a* − 97.63 × *Chlorophyll b*)/209 (4)

*Pigment content* (mg/g *fresh leaf*) = *Pigment concentration* (mg/L) × *V_extract_* (mL)/*fresh leaf weight* (g) (5)

### 2.5. Sample Digestion and Metal Content Determination

After harvest, the shoots were separated from the roots and dried in oven at 105 °C for 15 h. Approximately 0.05 g of dried shoots and 0.015 g of roots were subjected to acid digestion using nitric and hydrochloric acids 1:3 [62]. The dried biomass was mixed first with nitric acid and kept in dark for 48 h to avoid the excessive foam formation during heating. The digestion was performed at 90–95 °C on a hotplate for 2 h, the hydrochloric acid being added before heating the samples. The digestion samples were then placed into 10 mL (roots samples) and 25 mL (shoots samples) volumetric flasks and filtered using 0.45 μm PTFE filters. Metal concentrations in liquid samples were analyzed using an Atomic Absorption Spectrometer SensAA from GBC Scientific Equipment (GBC Scientific Equipment Pty Ltd, Braeside, Australia). Quality assurance and control (QA/QC) included a procedural blank, duplicate analysis and standard reference materials. The accuracy was calculated based on the relative error of the certified values of standard reference materials, and was less than 10%. The relative standard deviation (RSD) of duplicate samples was less than 5%.

### 2.6. Evaluation of Phytoremediation Performances

Pb(II) and Cd(II) phytoextraction/phytostabilization potential of *Brassica napus* L. was evaluated by calculating the following factors [63]:

Bioconcentration factor (BCF)

BCF = *C_metal in roots_*/*C_metal in soil_* (6)

Bioaccumulation coefficient (BAC)

BAC = *C_metal in shoots_*/*C_metal in soil_* (7)

Translocation factor (TF)

TF = *C_metal in shoots_*/*C_metal in roots_* (8)

Bioaccumulation factor is calculated by dividing the metal content in the aerial part of the plant (shoots and leaves) by the initial metal concentration in soil. Thus, BAC provides information about the ability of plant to accumulate metals in the aerial part of plant [64]. Bioconcentration factor gives information about the accumulation of metal from soil by roots. Translocation factor indicates the translocation of metals from the plant root into plant shoot. Values of TF less than one show that the plants accumulated metals in the roots and rhizomes more than in shoots or leaves, indicating an ineffective metal transfer from root to shoot [65]. Plants with BCF, BAF and TF values > 1, correlated with high biomass, high metal tolerance and accumulation, are considered phytoextractors, suitable for phytoextraction [1,66].

Based on the above factor values, it was established if *Brassica napus* L. is a hyper-accumulator, accumulator, bioindicator, excluder or a normal plant species for Pb(II) and Cd(II) [67,68,69].

## 3. Results

### 3.1. Phytotoxicity Test

#### 3.1.1. Cadmium and Lead Effects on *Brassica napus* L. Seeds Germination

**Figure 3:**
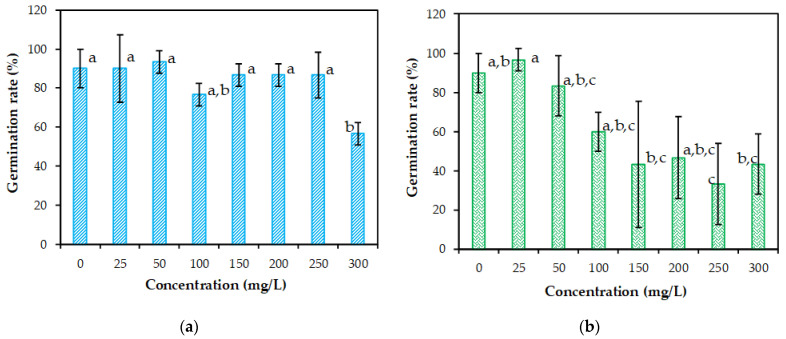
Seed germination rate of *Brassica napus* L. under (**a**) Pb(II) and (**b**) Cd(II) stress. Similar letters are statistically non-significant according to Tukey HSD test (*p* < 0.05). Means that do not share a letter are significantly different from each other, followed by letters that are later in the alphabet for lower means. Error bars indicate mean ± SD.

Figure 3 shows the effect of lead and cadmium ions on the germination of rapeseed under various metal concentrations. The seeds germinated in the case of all lead and cadmium treatments with solutions of 0 to 300 mg/L. At the highest concentration of lead treatment (300 mg/L of Pb(II) in the aqueous solution), *B. napus* exhibited the lowest germination degree (GD% of 56.67%), which is a significant reduction (*p* < 0.05) (Figure 3a). Based on grouping information using the Tukey method (Tukey pairwise comparisons), each of the concentration levels are associated with a grouping letter. According to our data, *group a* contains the statistical means of 0, 25, 50, 100, 150, 200 and 250 mg/L of Pb and *group b* contains the mean of 300 mg/L of Pb(II) (Figure 3a). The statistical mean of 100 mg/L Pb is included in both groups (a, b) (Figure 3a), which suggests that, at this concentration, the differences between the means are not statistically different. Means that do not share a letter, such as 300 mg/L Pb(II) and 0, 25, 50, 150, 200 and 250 mg/L Pb(II) are significantly different from each other (Figure 3a). In conclusion, we can say that the concentration of 50 mg/L Pb(II) resulted in the highest mean, followed by the means of 25, 0, 250, 200, 150, 100 and 300 mg/L Pb(II). The differences between the germination degree of the control and the treatments were not statistically significant, with the exception of the treatment with 300 mg/L Pb(II).

**Figure 4:**
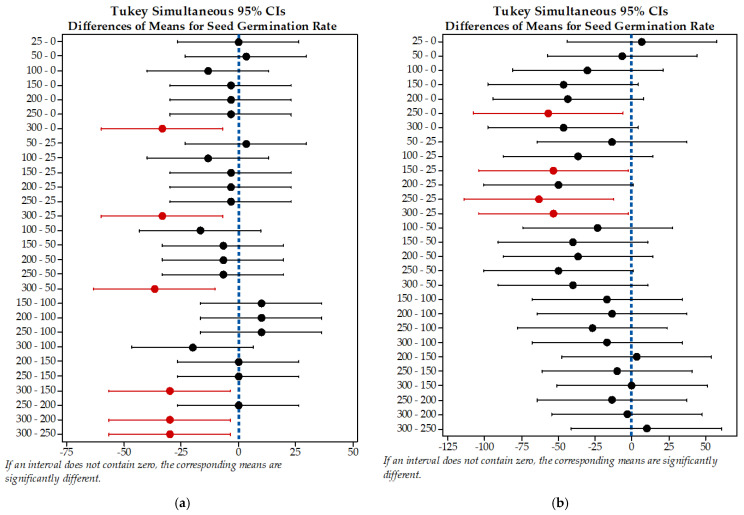
Tukey simultaneous 95% CIs differences in means for seed germination rate in presence of (**a**) lead and (**b**) cadmium (the intervals that do not contain zero are marked with red). The vertical blue dotted line indicates the point where the difference between the means is equal to zero, • estimated value of the difference between the means.

Further, the grouping results are included in a graph of intervals (Tukey simultaneous 95% CIs, where CI is the confidence interval). With our eight concentration levels, there are twenty-eight possible pairwise comparisons. The intervals of 300-0, 300-25, 300-50, 300-150, 300-200 and 300-250 mg/L Pb(II) do not contain zero; therefore, the difference between these means is statistically significant (*p* < 0.05) [70]. From these twenty-eight intervals, twenty-two include zero (pairwise containing intervals from 0 to 250 mg/L Pb(II), meaning that no statistical difference between the twenty-two means was supported (Figure 4a). Under the concentration range between 0 and 250 mg/L Pb(II), the germination degree was almost comparable with the control sample. This is supported by the fact that the confidence intervals for the pairs of means between 250 and 0 mg/L of Pb(II) all include zero (as mentioned above), confirming that the differences are not statistically significant (*p* > 0.05) [70].

In case of the treatments with cadmium ions, the germination degree showed a relatively low stimulation at 25 mg/L Cd(II) compared to the control sample (GD = 96.67% compared to GD = 90% related to control sample). Further, at concentrations between 50 and 300 mg/L of Cd(II), the percentage of germination rate for rapeseed was significantly reduced, since GD decreased from 83.33% to 43.33% (Figure 3b).

Taking into account the grouping information using the Tukey method, the means of 25 mg/L Cd(II) do not share all of the letters, indicating that a significant mean difference was identified. This is also available for the means of 250 mg/L Cd(II). Further, two groups (200 and 300 mg/L Cd(II)) share the same letters (b,c), whereas the other three groups (50, 100 and 200 mg/L Cd(II)) share the same letters (a,b,c), suggesting that the differences between the means are not statistically significant. Finally, we can say that the concentration of 25 mg/L Cd(II) resulted in the highest mean, followed by means of 0, 50, 100, 200, 300, 150 and 250 mg/L Cd(II), in that order. According to Tukey simultaneous 95% CIs, five intervals corresponding to 250-0, 150-25, 250-25 and 300-25 mg/L of Cd(II) do not contain zero; therefore, the difference between these means is statistically significant (*p* < 0.05). The rest of the intervals (twenty-three intervals) contain zero, suggesting that the corresponding means are not significantly different (*p* > 0.05) (Figure 4b).

#### 3.1.2. Cadmium and Lead Effects on *Brassica napus* L. Plant Growth

**Figure 5:**
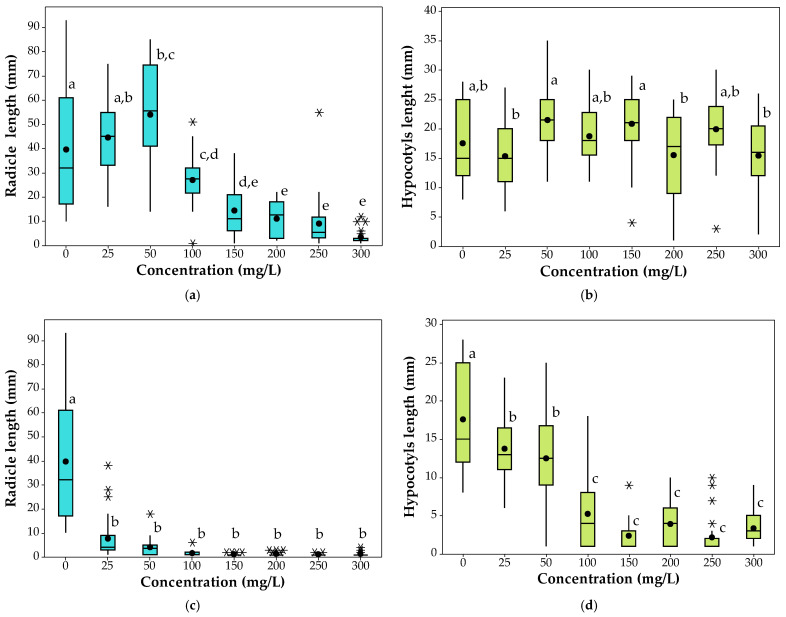
Box and whisker plots for (**a**) radicle and (**b**) hypocotyl length of *Brassica napus* L. at different levels of Pb(II) in solution and (**c**) radicle and (**d**) hypocotyl length at different levels of Cd(II) in solution. Box plots represent median (line inside each box) and the bottom and top of boxes represent the lower and upper quartiles, respectively. The mean is indicated with a (●) and the bottom and top of each whisker represent the minimum and maximum of each observed trait, respectively. (⚹) represents the outliers. Means that do not share a letter are significantly different, according to Tukey HSD test (*p* < 0.05).

Box and whisker plots were used to visualize the effects of different doses of cadmium and lead ions on the *Brassica napus* L. radicle and hypocotyl length at the end of the germination stage (Figure 5). In box and whisker plots, the median, extremes as the 1st and 3rd quartiles and the outlier values are highlighted. The radicle and hypocotyl mean length values of the control sample are 39.63 mm and 17.59 mm, respectively. According to the box and whisker plots, for the control sample, the root lengths of most seedlings are between 32 and 61 mm and the hypocotyl length is between 15 and 25 mm, with the median values being 32 mm and 15 mm, respectively. After exposure of the *Brassica napus* L. seeds at 25–300 mg/L of Pb(II), it was observed that, at 25 and 50 mg/L Pb(II), the mean of the radicle lengths is 12.62% and 36.08%, respectively, higher than the mean of the control sample, and, at concentrations higher than 100 mg /L Pb(II), the radicle length is 31.67–91.72% shorter (Figure 5a). The hypocotyl length of the plants grown in the presence of different doses of lead are shorter by a maximum of 12.62% or longer by a maximum of 22.23% compared to the control. According to Tukey test results, Pb(II) concentrations up to 300 mg/L do not significantly affect the length of the hypocotyls (Figure 5b). At Cd(II) concentration in the solution between 25 and 300 mg/L, the plant radicle lengths of the seedlings are significantly shorter than those of the control sample, the mean values being lower by 80.67–97.30% (Figure 5c). The hypocotyl lengths of the seedlings are shorter than the control sample by 21.77–87.61% (Figure 5d). The results of the Tukey test show that the mean of the radicle and hypocotyl lengths of the seedlings are significantly different compared to the mean of the control, even at low concentrations.

**Figure 6:**
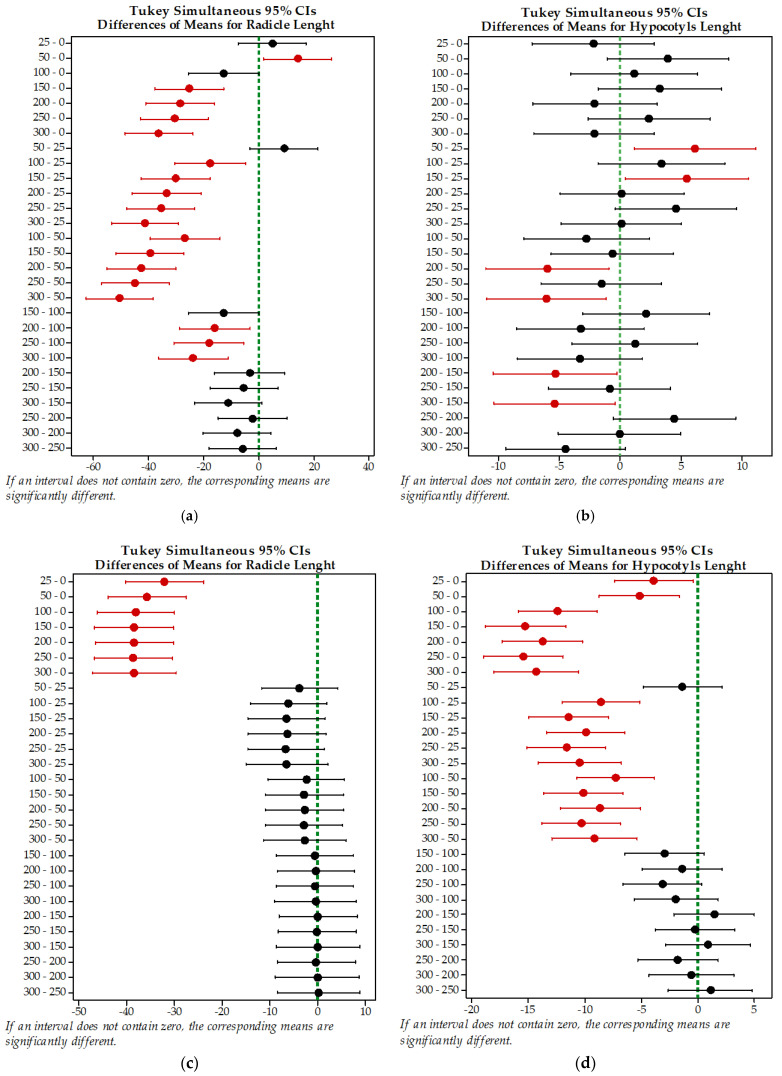
Tukey simultaneous 95% CIs differences in means for *Brassica napus* L: (**a**) radicle and (**b**) hypocotyl length at different levels of Pb(II) in solution and (**c**) radicle and (**d**) hypocotyl length at different levels of Cd(II) in solution (the intervals that do not contain zero are marked with red). The vertical green dotted line indicates the point where the difference between the means is equal to zero, • estimated value of the difference between the means.

The differences between the radicle and hypocotyl means were graphically represented in Figure 6. According to Tukey simultaneous 95% CIs, between the mean radicle length of the control seedlings and the mean at 50, 150, 200, 250 and 300 mg /L of Pb(II), there are statistically significant differences (Figure 6a). Furthermore, Figure 6b highlights that there are significant differences only between the pairs 50-25, 150-25, 200-50, 300-50, 200-150 and 300-150 mg/L Pb(II). In the case of the cadmium treatments, the Tukey simultaneous 95% CIs graphs (Figure 6c,d) show that there are significant differences between the roots length of the control and the roots length of the seedling grown with cadmium, but no differences between the other pairs were observed. The variation of cadmium ion concentrations between 25 and 300 mg/L affected not only the length of the roots but also the hypocotyl length of the seedlings, meaning that the length of the control sample is significantly different from all of the others. In addition, the hypocotyl mean length at 25 and 50 mg/L of Cd(II) are significantly different compared to the seedlings grown at higher concentrations (Figure 6d).

### 3.2. Plant Growth in Contaminated Soil

**Figure 7:**
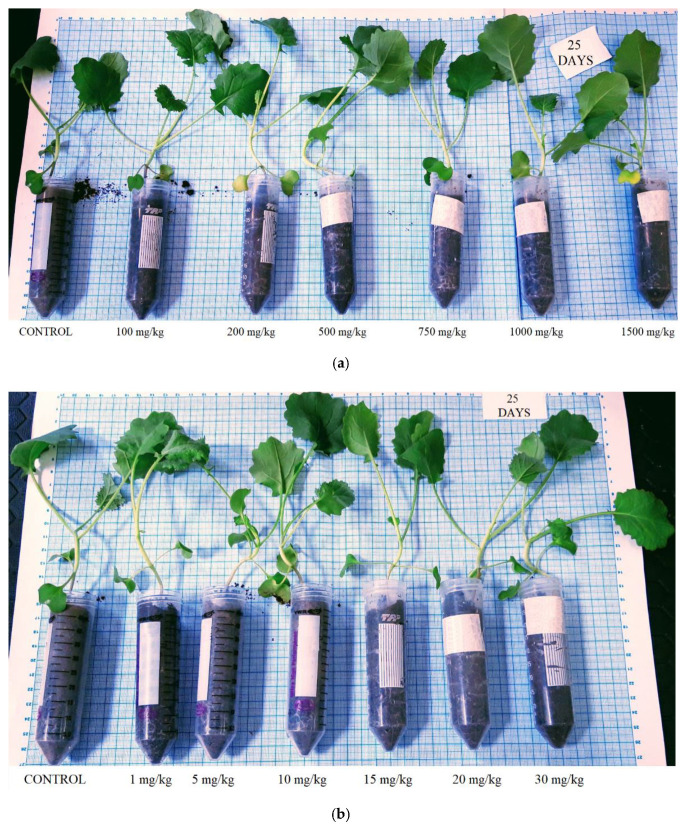
*Brassica napus* L. samples after 25 days of growing in the presence of lead (**a**) and cadmium (**b**).

In Figure 7, *Brassica napus* L. plants are presented at the end of the experiment after 25 days of growth in soil contaminated with lead (Figure 7a) and cadmium (Figure 7b). In all of the sample tubes, the seed fully germinated after 3 days of sowing. The aerial part of the plants cultivated in the lead-contaminated soil slowly exceeded the height of the control sample (with 2–3 cm) under the concentration range of 100 to 500 mg/kg Pb(II). At higher concentrations (750–1500 mg/kg Pb(II)), the plant height started to show a slow decrease (Figure 7a). In the presence of cadmium in soil under the studied treatments (1–30 mg/kg Cd(II)), no significant changes were observed in the aerial part of the plants compared to the control sample (Figure 7b).

#### 3.2.1. Lead and Cadmium Effects on the Contents of Photosynthetic Pigments

**Figure 8:**
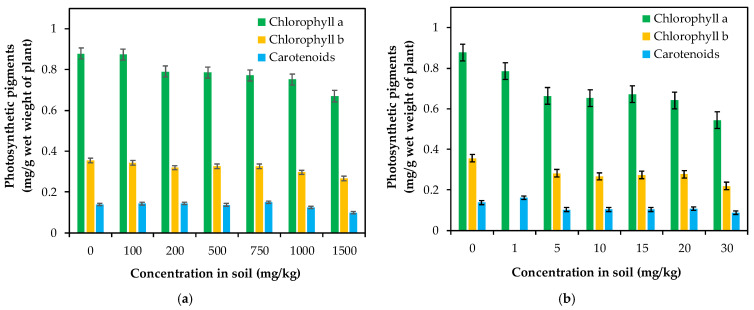
(**a**) Lead and (**b**) cadmium effects on the contents of photosynthetic pigments. Error bars indicate mean ± SD.

In order to assess the *Brassica napus* potential in the phytoremediation of contaminated soil, the amount of main photosynthetic pigments was determined. The experimental results described in Figure 8a,b reveal that the plants exposed for 25 days to different metal ion concentrations showed important changes in the chlorophyll a/chlorophyll b ratio, total chlorophyll and carotenoids content. The photosynthetic rate decreased with an increase in the Pb(II) and Cd(II) concentrations in the range of 100 to 1500 mg/kg soil, and from 1 to 30 mg/kg, respectively. Chlorophylls a (Chl a) and b (Chl b) control the photosynthetic potential of plants by capturing light energy from the sun, and represent the most important groups of photosynthetic pigments.

At low concentrations of lead, there is no change in the synthesis of chlorophyll pigments in rapeseed leaves, and the amount of pigments is equal to the control samples (Figure 8a), indicating a good plant adaptability. A slight decrease in the chlorophyll content was determined when the lead amount in the soil increased from 100 to 200 mg/kg, and then their profile slowly decreased. Thus, compared to the control plants, the chlorophyll a content decreased by 23.68%, the amount of chlorophyll b was reduced by 25%, and the carotenoids content decreased by 29.03% in plants exposed to a concentration of 1500 mg /kg of Pb(II) in soil. These results are correlated with previous studies [71,72], which reported a slight decrease in the chlorophyll pigment content and a good tolerance of lead ions by rapeseed plants. In addition, the chlorophyll a and b content decreased in comparison with the control samples (Figure 8b), and the degree of diminishing is similar for both pigments: in the case of chlorophyll a, the decrease was by 37.97%, and for chlorophyll b, the reduction was by 38.27 % for the maximum value of Cd ions, indicating that changes in the total chlorophyll content are the consequence of altering the whole growing process.

Likewise, it was found that the level of carotenoid pigments has been altered by the presence of Cd ions in the growing environment of *Brassica napus* plants. There was a quantitative decrease in the carotenoid pigments with an increase in the concentration of the contaminant, the effect being directly correlated with its dose: e.g., at concentrations of 5 mg/kg, the decrease was 22.51% for 20 mg /kg Cd(II) and the decrease continues up to 26.86%, whereas the trend is accentuated until 36% under a higher concentration of Cd(II) (30 mg/kg). However, in the range of the analyzed concentrations (1–30 mg/kg), there is no chlorosis effect on the leaves of rapeseed plants exposed to cadmium ions, which shows that the biosynthesis of chlorophyll pigments is not strongly inhibited [73].

**Figure 9:**
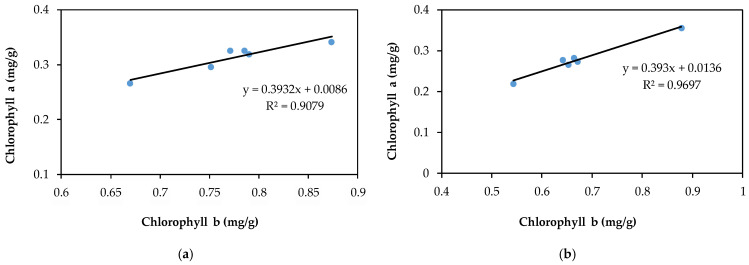
The linear relationship between chlorophyll a and b pigments in rapeseed leaves exposed to different concentrations of lead (**a**) and cadmium (**b**).

**Figure 10:**
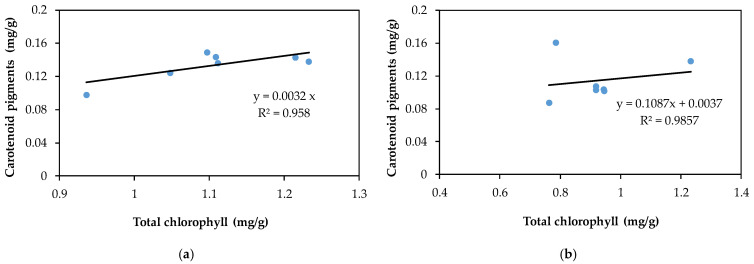
Relationship between total chlorophyll content and carotenoids in rapeseed leaves exposed to different concentrations of lead (**a**) and cadmium (**b**).

The relationship between Chl a and Chl b described in Figure 9a,b reflects a strong and positive linear relationship between Chl a and Chl b for both evaluated pollutants; the values of R^2^ are 0.969 for Cd(II) and 0.907 for Pb(II). The ratio of Chl a to Chl b slightly decreased in comparison to the control samples, but the value remained above 2. The ratio of Chl (a + b) to carotenoids for both pollutants was lower than for the control samples, indicating a stronger inhibition of chlorophyll synthesis in comparison with carotenoid pigments; the obtained values are over 7.3, which is normal for green plants that grow under low stress conditions [74]. Figure 10a,b illustrate the relationship between Chl (a + b) and Car, which is described by a linear equation, showing an insignificant decrease in carotenoids in correlation with the total changes in chlorophylls.

#### 3.2.2. Lead and Cadmium Accumulation in Root and Shoot of *Brassica napus* L.

**Figure 11:**
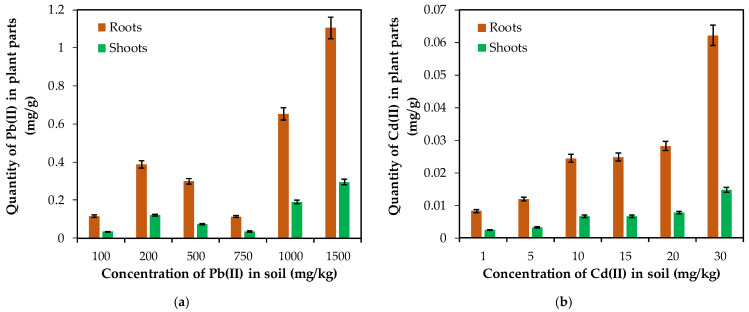
(**a**) Pb(II) and (**b**) Cd(II) content in *Brassica napus* L. tissues. Error bars indicate mean ± SD.

Concentration trends of lead and cadmium uptake among roots and shoots are presented in Figure 11. One of the most important parameter used to evaluate the potential of phytoextraction in plants is the total amount of metals accumulated in shoots. The increase in Pb(II) and Cd(II) accumulation in roots and shoots of *Brassica napus* L. plant varied with the respect to Pb(II) and Cd(II) treatments as related in Figure 11a,b respectively. 

The accumulation of Pb(II) in *Brassica napus* L. root ranged from 0.115 to 1.105 mg/g-plant, whereas the accumulation of Pb(II) by shoot ranged from 0.033 to 0.293 mg/g-plant. On the other hand, Cd(II) accumulation in *Brassica napus* L. root varied from 0.008 to 0.062 mg/g-plant, whereas the accumulation of Cd(II) by shoot ranged from 0.0024 to 0.014 mg/g-plant.

#### 3.2.3. Phytoremediation Potential of *Brassica napus* L.

Based on our results it was identified that *Brassica napus* L. is not a metal hyperaccumulator and thus is not suitable for phytoextraction. For both metals and for each treatment, TF < 1 indicates an ineffective metal transfer from root to shoot.

According to our experimental data, *Brassica napus* L. showed BCF values over 1 when the concentration in soil was 100 to 200 mg/kg Pb(II), whereas the BAC and TF values were < 1 in all cases of Pb(II) treatments, suggesting that *Brassica napus* L. is not an efficient plant for Pb transfer from root to shoot, but is yet suitable for Pb phytostabilization. At higher concentrations of Pb(II) in soil (500 to 1500 mg/kg Pb), the BCF, BAC and TF values are lower than 1 (Table 2 and Table 3). Considering the values of the BCF factor, which gives us information about the ability of the plant to accumulate the metal from soil by the roots, it was observed that, under treatments with 100 to 200 mg/kg Pb(II), *Brassica napus* L. is a lead accumulator, whereas, at higher metal concentrations (500 to 1500 mg/kg Pb(II)), it is an excluder plant. In conclusion, *Brassica napus* L. can be considered an adequate candidate for Pb accumulation and the phytostabilization (having a maximum value of BCF of 1.194) of contaminated soil, but under moderate concentrations of Pb in soil.

*Brassica napus* L. have the potential for Cd immobilization in roots, since BCF >1 and TF <1 for all treatments tested (1 to 30 mg/kg Cd(II)). Considering the BCF values (a maximum value of 8.297 and minimum of 2.073), we can conclude that *Brassica napus* L. may be a suitable candidate for Cd accumulation and the phytostabilization of contaminated soil.

## 4. Discussion

### 4.1. Lead and Cadmium Induced Phytotoxic Effects

In the current study, the germination percentage of *B. napus* L. seeds was significantly affected at 300 mg /L of Pb(II) compared to the control (*p* < 0.05). Further, a significant mean difference was identified at 25 mg /L of Cd(II). It is possible that, at this concentration, a relatively low stimulation of seeds occurs comparative to the control sample, whereas when at concentrations ranging from 50 to 300 mg/L of Cd(II), the germination degree reached a percentage of 43.33%. From this point of view, cadmium showed a higher toxic effect on seed germination compared to lead. In addition, the plant radicle and hypocotyl lengths of the seedlings seem to be stimulated under cadmium stress. It is obvious that the decrease in seed germination and seedling growth takes places along with increases in the metal dose, being the most sensitive part to Cd treatment. These results are in line with the results of other researchers [30,75,76]. For example, the order of toxicity for metal ions on the germination of *Pisum sativum* was Cd > Ni > Pb [77].

Different studies [46,74,78] reported that metal ions in toxic amounts generate a negative effect on the photosynthetic process. Our results indicated that the presence of cadmium and lead ions exerts a stress effect on rapeseed plants, inhibiting photosynthetic activity, which suggests a blockage of the electron transport chain and a damage to the antioxidant enzyme systems [79]. These effects could be explained by a deficiency of iron and zinc, the decrease in the amount of magnezium or by cadmium ions bonding to thiol groups in different enzymes [73,80]. Similar results [81] have been reported in the case of rapeseed plants exposed to Cd, where a reduction in the stomatal conductance and decrease in the photosynthetic rate were found. The effects of metals on the pigments synthesis were more pronounced in the case of cadmium compared to lead. In line with our results, Deswal and Laura [77] observed that the total amount of chlorophyll and carotenoids was reduced by the metals and that the order of toxicity is Cd > Ni > Pb.

Overall, the experimental results showed that cadmium and lead accumulation in soil inhibits seed germination and the growth in the length of the plant roots and stems. At the same time, the physiological processes of the plant are altered, such as the photosynthetic activity, and, consequently, the biological productivity is affected. Thus, at a decision-making level, it is imperative to develop and implement sustainable and efficient measures and programs applied in order to decrease the level of metal soil contamination, especially with cadmium and lead.

### 4.2. Phytoremediation Potential

Our results clearly indicated that metal bioaccumulation was higher in roots than in shoots, and that *Brassica napus* L. may be a suitable candidate for cadmium phytostabilization and soil conservation, at least for the specific soil used in our experiments. In the case of lead, the rapeseed behaved as an excluder plant at a higher contamination level of the soil. Pb is relatively insoluble in water and immobile, and thus the transport into the plant is low, mainly being concentrated in the plant roots [40]. Lead is considered to be generally blocked in roots as a non-essential element for plants, and is commonly not bioavailable for plants in contaminated soils due to its extreme insolubility, or in the normal range of soil pH compared with other metals, such as Zn, Cu or Cd [35,53,80]. Generally, the *Brassica* species showed a relative constant value of lead accumulation in different treatments of the soil contaminated with lead [35]. All of these data suggest the development of a lead exclusion mechanism by the plants (i.e., prevention of metal translocation from the roots to the shoots) [53]. On the other hand, different mechanisms, such as anatomical, biochemical and physiological, can be associated with metal translocation in plant, which may hinder the metal accumulation and distribution in aboveground tissues of the plant [82].

Our findings are in line with other results from literature [35,39,53,83]. Research performed by Drozdova et al. [53] found that six species of the Brassicaceae family had been uptake more Pb in roots than in leaves. From the fifteen Brassicaceae plant species collected from the Botanical Garden of St. Petersburg, Russia, only *Sinapis arvensis* and *Thlaspi arvense* were considered good candidates for Pb phytostabilization. According to their results, lead was the least mobile metal in soil, with low TF and BCF values compared to Zn, Cu and Cd. Angelova et al. [39] evaluated the efficiency of rapeseed for the phytoremediation of multimetal (Zn, Pb, Cd) contaminated soil in the presence and absence of organic soil amendments. In the absence of soil amendments, a considerable amount of metals (especially Pb) are accumulated in the roots, which is consistent with our results and other authors’ results, indicating that the translocation in the aerial parts of the plant is very limited (TF < 1). Grabner et al. [84], when testing the bioaccumulation capacity of five species of the Brassicaceae family (typical to the Slovenia region) of lead, cadmium and zinc, found that none of them could be considered hyperaccumulators plants. They can be used as biomonitors of metals (including two hybrids of *Brassica napus*).

It can be anticipated that *B. napus* that grows for a long period of time would produce a greater biomass and would have finally accumulated more Cd and Pb, increasing metal removal from the soil. Further, the use of Brassica in field conditions would have an advantage over other plants (e.g., *Thlaspi* plants), since they are known to be relatively easier to harvest [85]. The ability of rapeseed to retain Cd and Pb in their roots can contribute to a reduced accumulation of Cd and Pb in the crop plant, which may be of great interest to human health, especially when it is used for the production of vegetable oil for human nutrition. However, the uptake of metals and the translocation in different parts of the plant, as well as the degree of their tolerance, depend on the metal species and their concentrations in soil, ions bioavailability, plant species and their metabolism [86].

Overall, it is important to mention that the majority of the research is developed in pot experiments under controlled laboratory or greenhouse conditions. It is important to take into account that the final results can be very different compared to experiments performed in field conditions. Finally, it is clear that further studies are needed in this area in order to take into account the real conditions in screening plant varieties suitable for the phytoremediation of contaminated soils.

## 5. Conclusions

Our results led to the following final conclusions:

- The first negative effects of metal stress on *Brassica napus* L. were seen during the germination test performed in Petri dishes: at the highest concentration of the lead and cadmium treatment (with solutions of 300 mg/L), *B. napus* L. performed the lowest germination degree (a GD% of 56.67% and 43.33%, respectively) compared to the control sample;
- At concentrations of Cd(II) in the solution between 25 and 300 mg/L, the plant radicle and hypocotyl lengths of the seedlings are significantly shorter than those of the control sample, whereas, in the case of the lead treatments, it was observed that, at lower concentrations, the mean of the radicle and hypocotyl lengths are higher than the mean of the control sample, and start to decrease at concentrations of treating solutions- higher than 100 mg/L Pb(II);
- In soil pot experiments, the length of the plant was not significantly affected under the stress of metals. However, when testing lead and cadmium effects on the contents of photosynthetic pigments, we observed that important changes in the chlorophyll a/chlorophyll b ratio, total chlorophyll and carotenoids content appeared. We also observed a slight decrease in the chlorophyll pigment content and a good tolerance of lead ions by rapeseed plants. This was not the same in the case of the cadmium treatment, since a more evident quantitative decrease in the chlorophyll and carotenoid pigments with an increase in the concentration of the contaminant was obtained. It seems that cadmium is more toxic to the plant compared to lead;
- The accumulation of Pb and Cd in *Brassica napus* L. roots and shoots increased, along with the increase in their concentration in soil. The accumulated concentration of lead and cadmium was higher in roots compared to shoots;
- For both metals and for each treatment, a TF < 1 indicates an ineffective metal transfer from root to shoot. This means that, under tested conditions, *Brassica napus* L. is not a metal hyperaccumulator and thus is not suitable for phytoextraction. However, rapeseed can be considered a tolerant plant and a good candidate for Pb and Cd accumulation and the phytostabilization of contaminated soil. The results are valid for the soil considered and under the experimental conditions adopted.

Finally, in this study, we provide some new evidence of the potential of *Brassica napus* L. for the phytoremediation of soils contaminated with lead and cadmium conducted under in laboratory conditions for 25 days of growth by taking into account the actual condition in the Romanian pollution region. Our results prove the ability of metal bioaccumulation in rapeseed roots. Essentially, through the accumulation of metals in roots, the mobility and availability of metals is limited in soil. This means that rapeseed may be used as both an accumulator and as an excluder plant for metal-polluted soils in order to improve soil conservation in Romania. Since the solubility of metals depends on the characteristics of the soils, in particular, the pH, organic matter content and clay content, it is important to mention that the generic indices (BAF, BAC, TF) are valid only for the specific soil considered under the experimental conditions adopted, and that results cannot be generalized for other soils of different natures. Further studies are needed to test the potential of rapeseed in the phytoremediation of different soil types (agricultural soils, urban soils, soil from different industrial or mining sites, etc.).

Overall, the results can be considered as preliminary ones, since the potential of rapeseed should also be investigated along the whole life cycle in different phonological stages, from a juvenile plant to a mature plant. The successful transposition of the phytoremediation process on industrial scale should involve experiments performed in field conditions. Further work will also consider the possibility of processing the resulting metal-polluted rapeseed biomass; for example, to produce biodiesel. Nevertheless, more studies are necessary in order to assess the phytoremediation potential of rapeseed, taking into account the metals frequency and level occurring in the soil, such as Cd and Pb.

## Figures and Tables

**Table 1:** Benchmarks for traces of chemical elements in the soil [48].

Metals	Normal Values (mg/kg)	Alert Thresholds		Intervention Thresholds	
		Sensitive	Less Sensitive	Sensitive	Less Sensitive
Cadmium	1	3	5	5	10
Lead	20	50	250	100	1000

**Table 2:** BCF, BAC and TF of *Brassica napus* L. at different levels of lead and cadmium in soil.

Metal Ion	Concentration in Soil (mg/kg)	BCF	BAC	TF
Pb(II)	100	1.154	0.338	0.293
	200	1.940	0.601	0.309
	500	0.597	0.148	0.247
	750	0.150	0.045	0.303
	1000	0.652	0.190	0.292
	1500	0.737	0.196	0.2676
Cd(II)	1	8.297	2.473	0.298
	5	2.400	0.643	0.268
	10	2.445	0.670	0.274
	15	1.654	0.442	0.267
	20	1.411	0.391	0.277
	30	2.073	0.492	0.237

**Table 3:** Results and interpretation of phytoremediation potential of *Brassica napus* L.

Pollutant	Concentration in Soil (mg/kg)	Factor	Interpretation *
Lead	100	BCF > 1, TF < 1	Potential phytostabilizers
		BAC: 0.1–1	Medium accumulator
		BCF: 1–10	Accumulator
	200	BCF > 1, TF < 1	Potential phytostabilizers
		BAC: 0.1–1	Medium accumulator
		BCF: 1–10	Accumulator
	500	BCF < 1, TF < 1	Not suitable for phytoextraction
		BAC: 0.1–1	Medium accumulator
		BCF < 1	Excluder
	750	BCF < 1, TF < 1	Not suitable for phytoextraction
		BAC: 0.01–0.1	Low accumulator
		BCF < 1	Excluder
	1000	BCF < 1, TF < 1	Not suitable for phytoextraction
		BAC: 0.1–1	Medium accumulator
		BCF < 1	Excluder
	1500	BCF < 1, TF < 1	Not suitable for phytoextraction
		BAC: 0.1–1	Medium accumulator
		BCF < 1	Excluder
Cadmium	1	BCF > 1, TF < 1	Potential phytostabilizers
		BAC: 1–10	High-accumulator
		BCF: 1–10	Accumulator
	5	BCF > 1, TF < 1	Potential phytostabilizers
		BAC: 0.1–1	Medium-accumulator
		BCF: 1–10	Accumulator
	10	BCF > 1, TF < 1	Potential phytostabilizers
		BAC: 0.1–1	Medium-accumulator
		BCF: 1–10	Accumulator
	15	BCF > 1, TF < 1	Potential phytostabilizers
		BAC: 0.1–1	Medium-accumulator
		BCF: 1–10	Accumulator
	20	BCF > 1, TF < 1	Potential phytostabilizers
		BAC: 0.1–1	Medium-accumulator
		BCF: 1–10	Accumulator
	30	BCF > 1, TF < 1	Potential phytostabilizers
		BAC: 0.1–1	Medium-accumulator
		BCF: 1–10	Accumulator

* Note: All of the considerations are valid for the specific soil used in the present study.

## Data Availability

Not applicable.
